# Lymphatic Filariasis Disseminating to the Upper Extremity

**DOI:** 10.1155/2014/985680

**Published:** 2014-02-19

**Authors:** Catherine Maldjian, Vineet Khanna, Bevan Tandon, Matthew Then, Mohamed Yassin, Richard Adam, Michael J. Klein

**Affiliations:** ^1^Department of Radiology, University of Pittsburgh Medical Center, Pittsburgh, PA 15213-2582, USA; ^2^Department of Pathology, University of Pittsburgh Medical Center, Pittsburgh, PA 15213-2582, USA; ^3^Department of Infectious Diseases, University of Pittsburgh Medical Center, Pittsburgh, PA 15213-2582, USA; ^4^Department of Pathology and Laboratory Medicine, Hospital for Special Surgery, New York, NY 10021, USA

## Abstract

Lymphatic filariasis is the most common cause of acquired lymphedema worldwide (Szuba and Rockson, 1998). It is endemic to tropical and subtropical regions, and its effects are devastating. With over 100 million infected persons, it ranks second only to leprosy as the leading cause of permanent and long-term disability. *Wuchereria bancrofti* is the etiologic agent in 90% of cases. There is a dearth of published MRI findings with pathologically proven active infections, making this entity even more of a diagnostic dilemma. Imaging may provide the first clue that one is dealing with a parasite and may facilitate proper treatment and containment of this disease. This is the first report of pathologic correlation with MRI findings in the extremity in active filariasis. The magnetic resonance images demonstrate an enhancing, infiltrative, mass-like appearance with partial encasement of vasculature that has not been previously described in filariasis. Low signal strands in T2-hyperintense dilated lymphatic channels are seen and may depict live adult worms. We hypothesize that the low signal strands correspond to the collagen rich acellular cuticle. This, in combination with the surrounding hyperintense T2 signal, corresponding to a dilated lymphatic channel, may provide more specific MRI findings for active nematodal infection, which can prompt early biopsy, pathological correlation, and diagnosis.

## 1. Case Report

**Figure 1:**
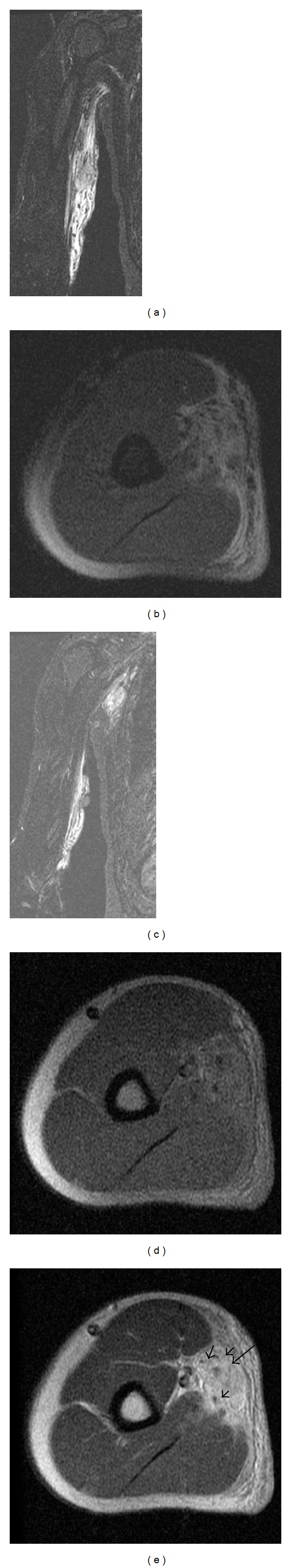
(a) Coronal STIR MR image of the right upper arm (TR = 3100; TE = 62.24; FOV = 38 cm). There is increased T2 signal extending from the axilla along the medial soft tissues of the upper arm following the lymphatic structures and paralleling the neurovascular bundle. (b) Axial T2 TSFSE (TR = 3000; TE = 42.816; FOV = 16) at the level of the mid to distal humerus where focal soft tissue swelling is present. There is an irregular area of increased T2 signal medially containing punctate low signal foci. (c) Coronal STIR MR image (TR = 3100; TE = 62.24; FOV = 38 cm). Axillary lymphadenopathy is demonstrated. (d) Axial precontrast T1WI (TR = 600; TE = 14.768; FOV = 16) at the level of the mid to distal upper arm demonstrates soft tissue infiltration medially. (e) Axial postcontrast T1WI (TR = 600; TE = 14.768; FOV = 16) at the mid to distal upper arm at the level of the swelling. There is enhancement medially with infiltrative appearance, closely abutting the brachial neurovascular bundle. Punctate and curvilinear low signal foci (arrows) are seen in the enhancing region.

A 33-year-old male from Nepal, who immigrated to the United States 3 years ago, presented to the Emergency Department with pain and redness at his right mid-arm for 10 days. He did not appear ill and had no fever or weight loss. The area was red, swollen, and tender. There was also enlargement of axillary lymph nodes. MRI demonstrated the presence of an enhancing soft tissue mass with infiltrative features, partially encasing the brachial vessels, in addition to the axillary lymphadenopathy (Figure 1). Foci of low signal intensity were also noted on both T2 and T1 weighted images (Figure 1).

**Figure 2:**
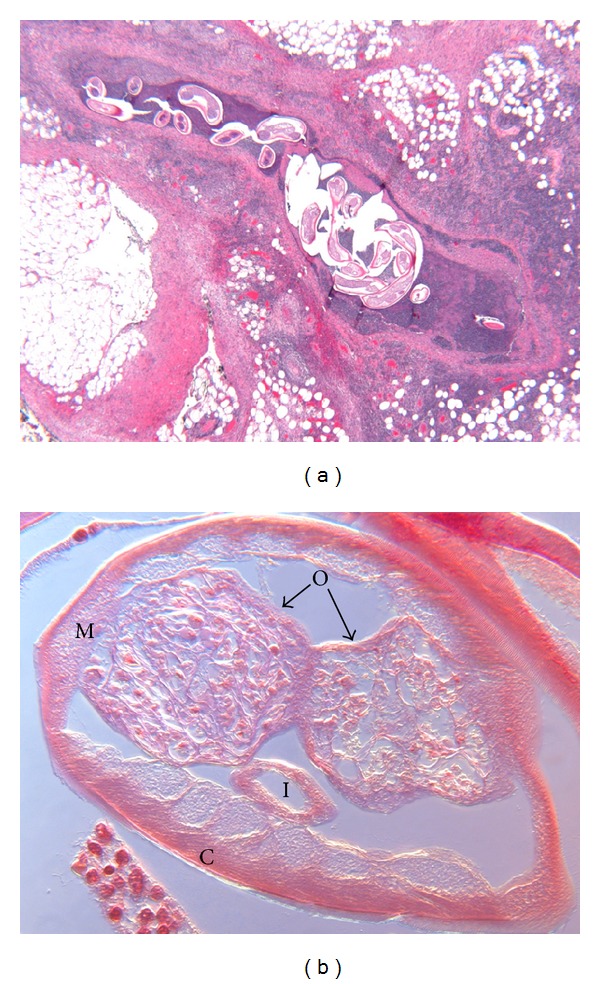
(a) Filariasis, lymphatic channel in perinodal adipose tissue. The lymphatic channel contains ensheathed helminthic structures consistent with an adult female *Wuchereria bancrofti* consisting of an external cuticle and a coelomic cavity containing paired ovaries and intestinal structures. The lymphatic channel shows perivascular fibrosis and contains within its both wall and lumen an inflammatory infiltrate composed primarily of lymphocytes and histiocytes. Eosinophils are rare, but individual cells cannot be discerned at this power. The inflammatory infiltrate and fibrous tissue extend into the perilymphatic adipose tissue (hematoxylin and eosin, ×25). (b) *Wuchereria bancrofti*. The external cuticle (C) appears focally striate, the muscle (M), intestine (I), and paired ovaries (O) are clearly visible, and the ovaries do not contain microfilariae, indicating a nongravid female (H&E, ×787, interference contrast with vertical image stacking).

An excisional biopsy of the medial arm mass revealed rubbery masses not attached to the major artery or vein. These masses measured 4 × 3 × 2.5 cm in toto. The pathological findings were consistent with microfilaria (Figure 2). Prominent endolymphatic permeation and distension by ensheathed parasitic structures were noted. The larval sheath demonstrated a thick cuticle and the terminal nuclear column not extending into the caudate space (Figure 2). The coelomic cavity contained two ovaries and was otherwise devoid of microfilariae, consistent with a nongravid female (Figure 2). The surrounding lymph node tissue demonstrated predominantly small lymphocytes with some arterial vascular ectasia and congestion. Serology for filaria (IgG_4_) was negative. Consultation with The Center for Diseases Control and Prevention (CDC) confirmed the presence of *Wuchereria bancrofti *nongravid adult female. Therapy with diethylcarbamazine (DEC) to be given for 12 consecutive days was recommended by the CDC. The patient remains in good condition with no new problems after 3 months of follow-up.

## 2. Discussion

Lymphatic filariasis is endemic to tropical and subtropical regions where it is the most common cause of acquired lymphedema [1]. Chronic infestation causes elephantiasis, a disfiguring disease. In 1997, the World Health Organization launched a campaign to eradicate lymphatic filariasis, which they identified as the second leading cause of permanent and long-term disability worldwide after leprosy. Today, over 120 million people are infected.

Mosquitoes serve as the vector and ingest microfilaria from the blood of an infected human host. Microfilaria develop into filariform larvae which are transmitted by insect bite to a human host. The offending organism in human lymphatic filariasis is *Wuchereria bancrofti*, *Brugia malayi*, or *Brugia timori*. Of these, *W. bancrofti* is the most prevalent species. These species are included under the phylum Nematoda, deriving from the Greek word nema and literally meaning thread, aptly describing its long, slender physique. The epidermis is not composed of cells, but rather an amalgam of cellular material and nuclei without organized structure or cell membranes. The epidermis secretes a tough outer shell or cuticle which is shed several times during maturation to adulthood. The cuticle serves as an exoskeleton, similar to its arthropod relatives. Arthropods, priapulids, and nematodes have been classified under a newly formed group called Ecdysozoa. Muscles under the epidermis of the nematode are oriented longitudinally, thus enabling the creature to bend only from side to side, but not allowing crawling, lifting, or more complex motion. A dorsal nerve, a ventral nerve, and a nerve ring connecting the two innervate the muscles. A gut cavity occupies the center of the organism.

There are two prior reports of MRI findings in the lymphatic system and one prior report of MRI findings in a joint [2–4]. In one report, nonenhancing fluid attenuation tubular structures in the chest, abdomen, and pelvis were noted, consistent with diffuse lymphangiectasia without discrete mass [3]. Cystic lymph node enlargement in the neck was described in another report with enhancement after contrast [2]. The third report describes involvement in an extremity consisting of filaremic arthropathy of the ankle [4]. Findings included inflammatory stranding in the subcutaneous fat with skin thickening, patchy marrow edema, and tenosynovitis. The patient described had elevated antibody level that confirmed exposure but did not necessitate active infection and does not identify the specific species. The diagnosis of active infection was presumed based upon the history and physical examination and relief of pain with 3 doses of diethylcarbamazine. The case we present is the first reported case with MRI findings in an extremity showing pathologic proof of live infection and the MRI findings are distinctly different than previous reports. The MRI demonstrated an enhancing area of soft tissue infiltration partially encasing the brachial vessels. The MRI also demonstrated linear tracts of increased T2 signal extending superiorly along the neurovascular bundle to merge with enlarged lymph nodes in the axilla, corresponding to dilated lymphatic channels.

Both neoplastic and infectious etiologies can be considered in the differential diagnosis. The propensity to spread along lymphatic channels has been demonstrated on imaging of filariasis [3]. The constellation of findings consisting of enhancing, infiltrative mass-like appearance with partial encasement of vasculature has not been previously described. The foci of diminished signal within these regions of dilated T2 hyperintense lymphatic channels may depict active nests of live adult worms. While not being previously described as such in humans, this description has been reported in ferrets [5]. Punctate low signal intensity centers within high signal lymph nodes have been described in nonhuman infection in ferrets and are thought to represent nests of viable adult nematodes in tortuous lymphatics and nodes, which was further supported by light scanning microscopy [5]. Acellular collagen is known to confer low signal on MRI [6]. We hypothesize that these foci of low signal reflect the exoskeleton of the nematode, which is derived of a collagen-rich extracellular matrix [7].

In summary, we present the first pathologically proven case of filariasis of the extremity with MRI findings. Imaging findings included enhancing, infiltrative soft tissue mass with encasement of the brachial vessels. Foci of diminished signal within the T2 hyperintense lymphatic channels were also seen and may provide a more specific finding for the diagnosis of filariasis. We hypothesize that these low signal intensity foci represent collagen-rich extracellular matrix which forms the thick cuticle or exoskeleton of the nematode. Imaging may provide the first clue that one is dealing with a live parasitic infection and may prompt early biopsy and definitive pathologic proof, thus facilitating proper treatment and containment of this disease.
